# Introduction to the special issue: Recap of ninth International Conference on Health Policy Statistics

**DOI:** 10.1007/s10742-012-0096-8

**Published:** 2012-06-07

**Authors:** Thomas E. Love, A. James O’Malley

**Affiliations:** 1 Case Western Reserve University, Cleveland, OH USA; 2 Harvard Medical School, Boston, MA USA

The papers in this special double-issue of *Health Services and Outcomes Research Methodology* are based on presentations at the ninth International Conference on Health Policy Statistics (ICHPS) held October 5–7, 2011 at The Ritz Carlton in Cleveland, OH. We are very excited to publish nine papers that are not only of the highest quality but also serve as a permanent reminder of the very high standard of research on display at the conference.

More than 210 statisticians, methodologists, and health policy experts from all over the world gathered for the ninth ICHPS—a biennial meeting jointly sponsored by the ASA and its Health Policy Statistics Section. Conference Co-Chairs James O’Malley (Harvard University) and Thomas Love (Case Western Reserve University) were ably supported by a 29-member Scientific Organizing Committee and 10-member Advisory Board.

ICHPS 2011 was made possible by the Agency for Healthcare Research and Quality (AHRQ), through an R-13 grant. Project Officer Melford Henderson took an active role, challenging attendees to find effective mechanisms for disseminating results so that they translate into actual policy and practice. This special issue of *Health Services and Outcomes Research Methodology*, is one avenue of outreach emanating from the conference.

The meeting’s theme—Advancing Methods to Improve Health Care—reflected the growing importance of research methods in facilitating informed discussions regarding health reform and other efforts to improve health care in the United States. The newly minted Patient Centered Outcomes Research Institute’s calls for research reflect the impact of many of the methods showcased at ICHPS 2011.

As evidenced in the papers published in this issue, the presentations included a broad range of topics, including longitudinal data, spatial data, causal inference and treatment heterogeneity, quasi-experimental studies and novel use of randomization, and Bayesian analysis including prior specification, hierarchical modeling and random effects. Details of all sessions at the conference can be found at www.amstat.org/meetings/ichps/2011.

Rousing plenary addresses were delivered by Steven Nissen, M.D., Department Chair of Cardiovascular Medicine at the Cleveland Clinic on “The Drug Safety Wars”, and Arlene Ash, Ph.D., Chief of the Division of Biostatistics and Health Services Research in the Department of Quantitative Health Sciences at the University of Massachusetts Medical School on “Making Statistics Relevant: Why Not Change The World?” Dr. Nissen’s keynote described the discovery of safety concerns involving the pharmaceutical blockbusters Vioxx, Muraglitazar, and Avandia; the challenge of getting these concerns acknowledged; and subsequent attempted cover-ups. His talk empowered the audience to use rigorous statistical practice to maintain a high standard of integrity. Dr. Ash provided a wonderful description of her work in health policy statistics, offering sage advice and perceptive glimpses of the future. She encouraged the pursuit of problems of real interest, and emphasized the power of independent thought and action.

The conference also featured the presentation of the Health Policy Statistics Section’s biennial Awards. Honored with the Long-Term Excellence Award were Drs. Paula Diehr (University of Washington, emeritus) and Sharon-Lise Normand (Harvard University), while the Mid-Career Award was presented to Dr. James O’Malley (Harvard University). A Thursday evening visit to the House of Blues and Cleveland’s East Fourth Street district afforded opportunities to mingle in a relaxed environment while enjoying Cleveland’s hospitality and delicious food.

The eleven conference workshops (together attracting nearly 500 attendees) included sequences on causal inference and patient reported outcomes, plus workshops on quantile regression, multiple comparisons, network meta-analysis, and evaluating the accuracy of medical tests and biomarkers. Jeffrey Rhoades from AHRQ concluded the conference with an entertaining and informative introduction to the Medical Expenditure Panel Survey.

This ICHPS was delighted to provide 15 student travel awards, supported by multiple industry and academic partners, including Lilly USA, Harvard Clinical Research Institute (HCRI), Valence Health, Millennium Pharmaceuticals, and ASA’s Cleveland Chapter as well as Case Western Reserve University’s Department of Epidemiology & Biostatistics, the Cleveland Clinical and Translational Science Collaborative and the Center for Health Care Research and Policy. In addition, the Conference Chairs were grateful for ASA’s outstanding support, and in particular, for the stellar work of the conference’s planner, Lisette Werbowetzki.

We eagerly anticipate the tenth ICHPS, planned for October 2013. For more information, contact Conference Chairs Andrew Zhou (azhou@u.washington.edu) and Donald Hedeker (hedeker@uic.edu).

## List of papers in the special issue

The papers in this special issue are as follows:

1. Assessing the Sensitivity of Treatment Effect Estimates to Differential Follow-Op Rates: Implications for Translational Research by Beth Ann Griffin, Daniel McCaffrey, Rajeev Ramchand, Sarah Hunter, and Marika Suttorp (all RAND Corporation).
2. Bias and variance tradeoffs when combining propensity score weighting and regression: with an application to HIV status and homeless men by Daniela Golinelli (RAND), Greg Ridgeway (RAND), Harmony Rhoades (University of Southern California), Joan Tucker (RAND) and Suzanne Wenzel (USC).
3. Perils and Prospects of Using Aggregate Area Level Socioeconomic Information As a Proxy for Individual Level Socioeconomic Confounders in Instrumental Variables Regression by Yenchih Hsu, Scott Lorch, and Dylan Small (all University of Pennsylvania).
4. Assessing the Privacy of Randomized Vector valued Queries to a Database using the Area Under the Receiver-Operating Characteristic Curve by Gregory Matthews (University of Massachusetts) and Ofer Harel (University of Connecticut).
5. Degrees of health disparities: Health status disparities between young adults with high school diplomas, sub-baccalaureate degrees and baccalaureate degrees by Janet Rosenbaum (University of Maryland).
6. Clinically relevant graphical predictions from Bayesian joint longitudinal-survival models by Laura Hatfield (Harvard Medical School) and Bradley Carlin (University of Minnesota).
7. Joint Modeling of Longitudinal Outcomes and Survival in a Mesothelioma Trial by Ping Wang, Wei Shen, and Mark Boye (all Lilly, Inc.).
8. Mixed-effects regression modeling of real-time momentary pain assessments in Osteoarthritis (OA) patients by Cynthia Coffman, Kelli D. Allen and Robert F. Woolson (all from Durham VA Medical Center and Duke University).
9. Using AIC in the multiple linear regression framework with multiply imputed data by Ashok Chaurasia and Ofer Harel (both University of Connecticut).

We hope that you enjoy reading the papers in this special issue and that they may entice to you attend and contribute to future ICHPS meetings.

